# Influence of Matrix Type on Marginal Gap Formation of Deep Class II Bulk-Fill Composite Restorations

**DOI:** 10.3390/ijerph19094961

**Published:** 2022-04-19

**Authors:** Britta Hahn, Imme Haubitz, Ralf Krug, Gabriel Krastl, Sebastian Soliman

**Affiliations:** Department of Conservative Dentistry and Periodontology, University Hospital Würzburg, Pleicherwall 2, 97070 Würzburg, Germany; imme.haubitz@gmx.de (I.H.); krug_r@ukw.de (R.K.); krastl_g@ukw.de (G.K.); soliman_s@ukw.de (S.S.)

**Keywords:** transparent matrix, metal matrix, bulk-fill technique, centripetal technique, marginal gap formation, class II restoration, SEM

## Abstract

Background: To test the hypothesis that transparent matrices result in more continuous margins of bulk-fill composite (BFC) restorations than metal matrices. Methods: Forty standardized MOD cavities in human molars with cervical margins in enamel and dentin were created and randomly assigned to four restorative treatment protocols: conventional nanohybrid composite (NANO) restoration (Tetric EvoCeram, Ivoclar Vivadent, Schaan, Liechtenstein) with a metal matrix (NANO-METAL) versus transparent matrix (NANO-TRANS), and bulk-fill composite restoration (Tetric EvoCeram Bulk Fill, Ivoclar Vivadent, Schaan, Liechtenstein) with a metal matrix (BFC-METAL) versus transparent matrix (BFC-TRANS). After artificial aging (2500 thermal cycles), marginal quality was evaluated by scanning electron microscopy using the replica technique. Statistical analyses were performed using the Mann–Whitney U-test and Wilcoxon test. The level of significance was *p* < 0.05. Results: Metal matrices yielded significantly (*p* = 0.0011) more continuous margins (46.211%) than transparent matrices (27.073%). Differences in continuous margins between NANO (34.482%) and BFC (38.802%) were not significant (*p* = 0.56). Matrix type did not influence marginal gap formation in BFC (*p* = 0.27) but did in NANO restorations (*p* = 0.001). Conclusion: Metal matrices positively influence the marginal quality of class II composite restorations, especially in deep cavity areas. The bulk-fill composite seems to be less sensitive to the influence of factors such as light polymerization and matrix type.

## 1. Introduction

The impacts of various oral health conditions on oral health-related quality of life (OHRQoL) have been extensively studied in the literature [1]. It is well documented that higher DMFS (Decayed Missed Filled Surfaces) scores are associated with a significantly greater impact on self-reported OHRQoL than lower DMFS scores [2]. Thus, modern restorative dentistry should focus on prevention and high-quality, long-lasting restorations in order to slow down the “restorative death spiral”. In recent decades, considerable developments have been made in dental resin composites [3]. Bulk-fill composite (BFC) materials, in particular, have gained considerable clinical acceptance [4,5], because they enable the placement of thicker composite layers (~4 mm) with a sufficient depth of cure and less polymerization shrinkage stress [4,6,7,8]. A higher depth of cure has been achieved by using higher-translucency composite materials to improve light transmission or by adding optimized highly reactive photo-initiators such as a dibenzoyl germanium derivative (e.g., Ivocerin^®^ in Tetric EvoCeram^®^ Bulk Fill; Ivoclar Vivadent, Schaan, Liechtenstein), besides the conventional camphorquinone [9,10,11,12].

Bulk filling simplifies the restorative process, saves time, and reduces the risk of technical errors, such as the formation of voids between layers [12,13]. In view of their properties, it can be concluded that bulk-fill materials can be recommended for large and deep cavities [14,15]. In clinical practice, dentists are often confronted with cavities significantly deeper than 4 mm, which are especially demanding with regard to light polymerization. Furthermore, such deep defects are difficult to seal with a matrix and moisture control remains a major challenge. It has been shown that pre-contoured matrices are beneficial for creating proximal contacts [16,17,18,19], especially when combined with separation rings, and for reducing overhangs [20,21]. Flat matrix bands also produced satisfactory results in other studies [22,23,24,25,26]. When restoring deep cavities with margins below the cementoenamel junction (CEJ), rigid metallic matrices may facilitate matrix placement and adaptation [20,26,27,28,29,30]. However, light polymerization may be compromised if the light guide tip is partially covered when using a metal matrix [7,31]. On the other hand, an older study showed that metal matrices with a reflective surface can focus the light cervically within the cavity and thus achieve a higher depth of cure than transparent matrices [26]. Optimal positioning and angulation of the light guide tip is the key to ensuring light transmission to each area of the composite layer [30,32]. Accordingly, use of the three-sited light curing technique after metal matrix removal has been recommended to ensure a sufficient depth of cure [33,34]. Nevertheless, even this polymerization technique does not prevent the attenuation of light intensity during the penetration of dental hard tissue, so the extension of curing time also seems necessary [35,36].

Countless matrix systems are available on the market, including flat or pre-contoured bands, retainer-fixed circumferential systems, and sectional matrices, and most feature either metal or plastic matrices [17,18,20,21,22,37,38,39].

A recent survey by Schaalan [22] revealed that Egyptian dentists prefer sectional matrix systems over circumferential matrix systems, but the author did not mention whether there was a difference between plastic and metal matrices [22]. In a clinical trial by Demarco et al. [27], however, the clinical performance of composite restorations did not depend on whether a transparent plastic or metallic matrix was used, but rather was more strongly influenced by deterioration of the adhesive bond and composite material—a conventional micro-hybrid composite (Filtek P60, 3M ESPE, St. Paul, MN, USA) in this case. However, there are no studies investigating this question for bulk-fill materials, which are usually placed using the bulk-fill technique. It has been shown that restoring deep cavities leads to large volumes of composite material if filled in bulk, and that the larger the volume of composite material, the greater the marginal gap formation [40]. The current literature lacks information on the extent to which the type of matrix (transparent or metal) might influence marginal gap formation in deep class II bulk-fill composite restorations. Such data would be useful, since metal matrices are easier to place but can impair light polymerization, as described above. Therefore, this in vitro study aims to test the hypothesis that transparent matrices result in more continuous margins of bulk-fill composite restorations than metal matrices.

## 2. Materials and Methods

Ethical approval for the use of extracted human teeth for material testing of dental restorations was obtained from the local Ethics Committee (approval number: AZ 15/15). Forty freshly extracted, caries-free human molars of nearly equal size were stored in 0.1% chloramine T solution until further processing. All mesio-occlusal-distal (MOD) cavities were prepared and filled within seven consecutive days. The specimens were randomly assigned to four treatment groups of ten specimens each featuring two types of restorative materials and techniques—conventional nanohybrid composite (Tetric EvoCeram, Ivoclar Vivadent, Schaan, Liechtenstein) for centripetal layering versus bulk-fill composite (Tetric EvoCeram Bulk Fill, Ivoclar Vivadent, Schaan, Liechtenstein) for bulk-filling—and two types of matrix systems—metal (METAL) matrices versus transparent plastic matrix bands (TRANS) secured in a Tofflemire retainer, respectively. Self-curing resin (Paladur, Heraeus Kulzer, Hanau, Germany) was used to embed the teeth by means of a Teflon mold with the occlusal surfaces parallel to the ground.

**Figure 1:**
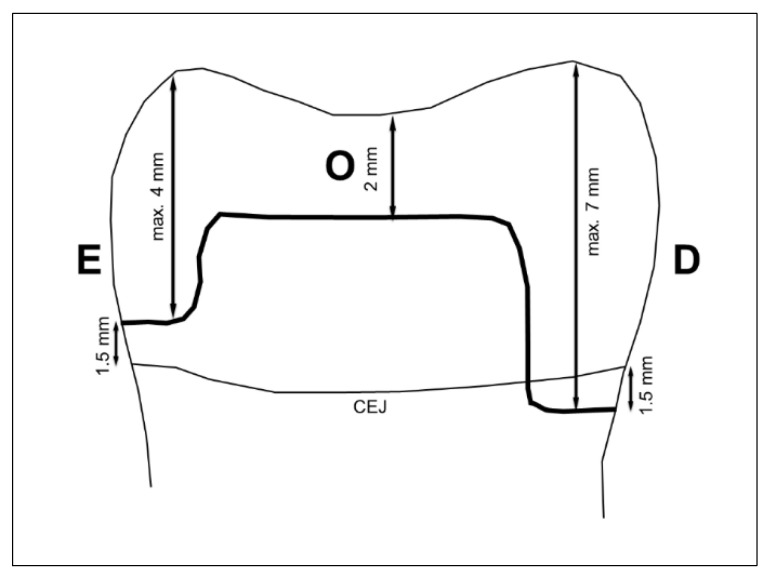
Cavity design and cavity dimensions (arrows); E = proximal box located within enamel; O = occlusal cavity; D = proximal box cervically located in dentin; CEJ = cementoenamel junction.

Box-shaped MOD cavities (occlusal box: 2.0 mm deep, 3.5 mm wide) were prepared using hand-held cylindrical 1.2 mm diamond burs (grain size 80–100 µm and 40 µm; Komet, Lemgo, Germany) in a high-speed contra-angle handpiece (INTRAmatic Lux 3 25 LH, KaVo, Biberach, Germany). Interproximal boxes were prepared using the same instrument to a buccolingual width of 3.5 mm. The cervical margin of the mesial box was located 1.5 mm above the cementoenamel-junction, but not deeper than 4.0 mm from the occlusal surface, and that of the distal box was located 1.5 mm beyond the CEJ, but not deeper than 7.0 mm from the occlusal surface. The enamel parts of the interproximal boxes were converted into a bevel design using a sonic preparation system (SONICflex LUX 2000 L, KaVo, Biberach, Germany) with a standardized oscillating diamond tip (SONICsys Approx, No. 36, KaVo, Biberach, Germany), which was completely immersed into the tooth. The beveled design in the enamel was finished with an oscillating Bevelshape file (No. 01, Intensiv, Montagnola, Switzerland) in a contra-angle handpiece (INTRAmatic Lux 2 20 KN, KaVo, Biberach, Germany) with an oscillating head (Intra EVA Head L6 R, KaVo, Biberach, Germany). The bevel width was 1 mm. The box-shaped design in dentin was finished with an oscillating Cavishape file (CS 140, Intensiv, Grancia, Switzerland). All preparation instruments were replaced with new instruments after ten completed cavity preparations. Cavity design is shown in Figure 1. Cavity dimensions were continuously monitored during preparation by means of loupes (2.5× magnification, Zeiss, Oberkochen, Germany) and a periodontal probe.

**Figure 2:**
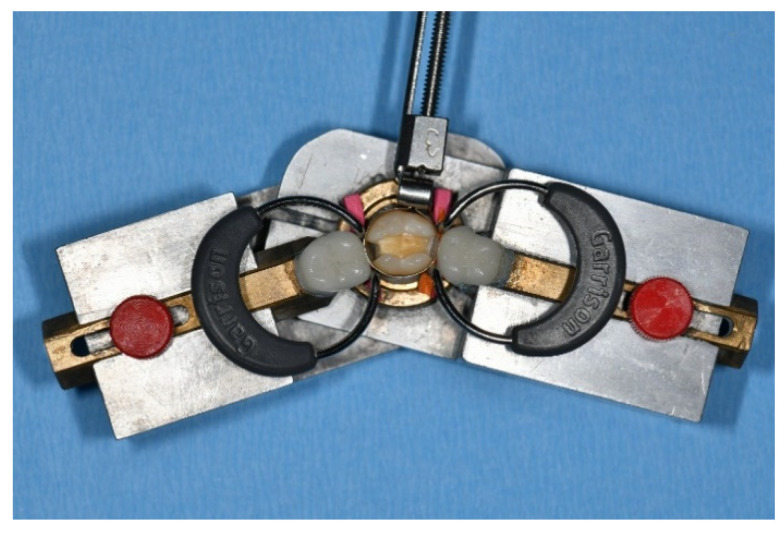
Artificial dental model with mounted specimen tooth, metal matrix secured in a Tofflemire holder, wooden wedges, and separation rings.

**Figure 3:**
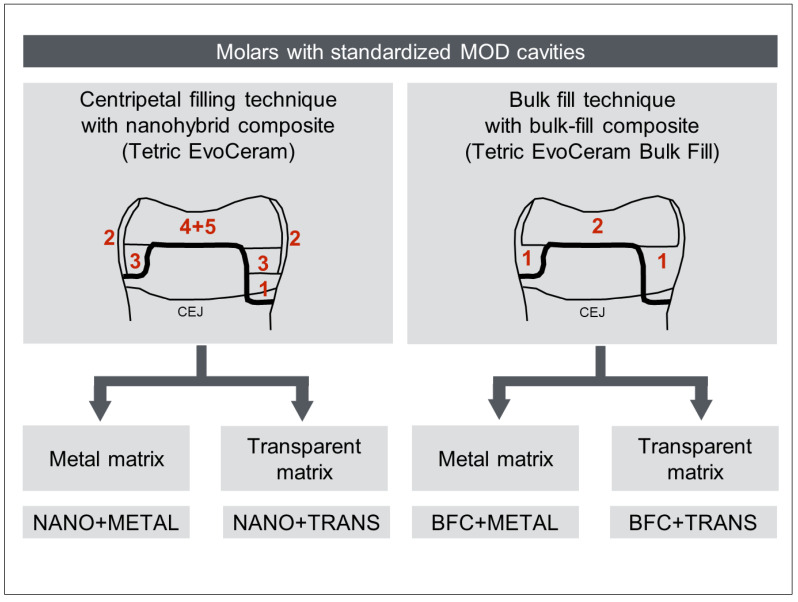
Experimental setup with the four experimental groups; red digits represent the order and number of composite increments. NANO = Tetric EvoCeram; BFC = Tetric EvoCeram Bulk Fill; METAL = metal matrix; TRANS = transparent matrix; CEJ = cementoenamel junction.

As shown in Figure 2, the test teeth were mounted between artificial tooth models to simulate physiological interproximal relations. The mounted specimens were restored using either metal matrix bands (399 C, Kerr, Bioggio, Switzerland) or transparent matrix bands (DEL, Dental Exports London, Feltham, UK), respectively, secured in a Tofflemire retainer (Omnident, Rodgau Nieder-Roden, Germany). Each matrix band was secured interdentally–cervically with wooden wedges (Hawe Sycamore Interdental Wedges, Kerr; Orange, CA, USA), and laterally, at the vertical cavity margins, with separation rings (Composi-Tight 3D 400 Thin Tine G/Ring, Garrison Dental Solutions, Spring Lake, MI, USA). The contact area was burnished with a hand instrument (PFI19, Hu-Friedy, Frankfurt, Germany) so that no visual space was left between the matrix and the adjacent tooth. Enamel and dentin were etched (30 and 15 s, respectively) with 37% phosphoric acid gel (Omni-Etch, Omnident, Rodgau, Germany) and then rinsed with water spray for 20 s. A two-step etch-and-rinse bonding agent (OptiBond FL, Kerr Italia S.r.l., Scafati, Italy) was applied and processed according to the manufacturer’s instructions. Bonding agent was polymerized from the occlusal direction, and each proximal box was light-cured for 20 s. Cavities were filled with conventional nano-hybrid composite (Tetric EvoCeram, Ivoclar Vivadent, Schaan, Liechtenstein) using a centripetal layering technique or with bulk-fill composite (Tetric EvoCeram Bulk Fill, Ivoclar Vivadent, Schaan Liechtenstein) using a bulk-fill technique (see Table 1). The centripetal layering technique involves initial restoration of the absent proximal wall, thus transforming the class II cavity into a class I cavity. Each increment of composite was light-cured for 20 s with a mono-wave LED light curing device (Elipar Freelight 2, 3 M ESPE, Seefeld, Germany) at 1020 mW/cm^2^, verified with a radiometer (Bluephase Meter II, Ivoclar Vivadent, Schaan, Liechtenstein). With the bulk-fill technique, intermediate light-curing was performed once after filling the proximal boxes and modeling the proximal wall, as otherwise, the maximum increment thickness of 4 mm would have been significantly exceeded. With the centripetal technique, on the other hand, intermediate light-curing was performed after each individual increment. After removal of the matrix band, restorations were post-cured for a further 20 s from the buccal and lingual side, respectively, with the specimen teeth still secured within the artificial tooth model. An overview of the experimental groups and restorative techniques is given in Figure 3.

The test teeth were then taken off the artificial tooth model for hand-held finishing. Composite overhangs were removed with a scalpel (No. 15, Braun, Aesculap AG, Tuttlingen, Germany), and the restorations were finished with a brown rubber polisher (Komet, Lemgo, Germany) at 10,000 rpm with water spray cooling to allow SEM analysis of the restoration margins. All restorations and measurements were performed by one calibrated operator (B.H.) after the samples were blinded by an independent observer (S.S.).

For artificial aging, the specimens were stored in physiological saline solution in an incubator (Memmert, Schwabach, Germany) for seven days at 37 °C followed by thermal cycling (MT & UKT 600, Lauda, Lauda Königshofen, Germany). The specimens were subjected to 2500 cycles of alternating cold and hot water treatment (5 °C and 55 °C) following another seven days of storage in physiological saline solution.

**Figure 4:**
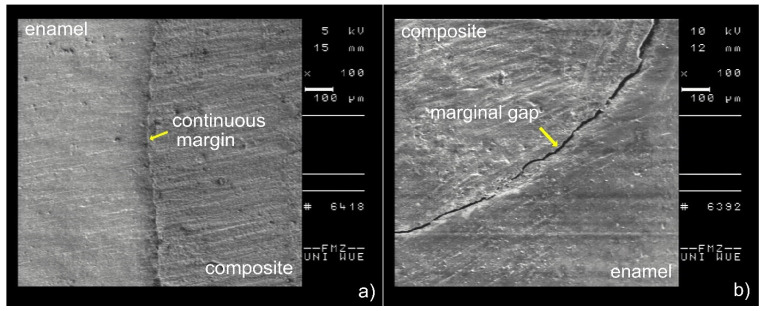
Representative SEM images of the marginal quality outcomes: (**a**) continuous margin and (**b**) marginal gap.

The specimen teeth (*n* = 40) were replicated with epoxy resin (Araldite, Ciba-Geigy, Basel, Switzerland) for analysis by scanning electron microscopy (SEM). The mesial and distal surfaces of each specimen were cast with silicone, yielding a total of *n* = 80 replicas. These were subsequently sputter-coated with gold in a sputter coater (EMITECH K550 Emitech, Taunusstein, Germany). Marginal quality was assessed by measuring the percentage of continuous margins and marginal gaps, respectively, using a scanning electron microscope (DSM 940, Zeiss, Oberkochen, Germany) with 100× to 1000× magnification and calibrated measuring software (RaEm^©^; programmer: Peter Müller, 97267 Himmelstadt, Germany). The results were expressed as a percentage of the respective quality outcome variables along the total margin length for each test group. The two different marginal qualities (continuous margin vs. marginal gap) are illustrated in Figure 4. For clarity, only the proportion of continuous margins [%] is depicted in the results section. Therefore, the proportion of marginal gaps is 100% minus the proportion of continuous margins.

All statistical analyses were performed using the WinMEDAS statistical software package (Version 8/20, C. Grund, Würzburg, Germany). Since there was no Gaussian normal distribution of the measured values, rank tests were used. The Wilcoxon test (*p*-values depicted as P_w_) was used for comparison between two measurements of dependent samples, i.e., to test for differences between enamel and dentin margins. The Mann–Whitney U-test (*p*-values depicted as P_u_) was used for independent samples to compare measurements between the two composite materials or the two matrix systems, respectively. In case of statistically significant differences, Cohen’s effect size (ES d*_Cohen_*) was calculated. Cohen’s effect size shows how strongly a parameter affects the outcome and reflects its clinical relevance. The effect sizes were classified as small (ES d*_Cohen_* < 0.5), medium (ES d*_Cohen_* = 0.5–0.8) or large (ES d*_Cohen_* > 0.8). To compare the test results quantitatively, *p*-values were calculated. The significance level was set at *p* < 0.05. *p*-values were marked with asterisks to denote the significance level as follows: * *p* < 0.05, ** *p* < 0.01, *** *p* < 0.001.

## 3. Results

### SEM Analysis

**Figure 5:**
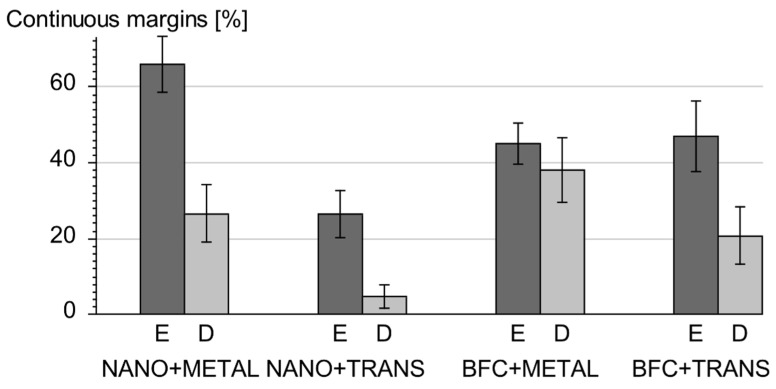
Mean percentages with standard deviation of continuous margins in enamel (E) and dentin (D) in all groups; NANO = Tetric EvoCeram; BFC = Tetric EvoCeram Bulk Fill; METAL = metal matrix band, TRANS = transparent matrix band.

Figure 5 shows the proportions of continuous margins [%] in enamel and dentin in all groups. The percentage of continuous margins was significantly higher in cavity segments located in enamel than in dentin in all four test groups (Table 2 and Figure 5; P_w_ = 0.00005 ***). Metal matrices yielded significantly more continuous margins than transparent matrices (P_u_ = 0.0011 **; Table 3, line 3) with a large effect size in dentin (ES d*_Cohen_* = 0.87; Table 3, line 2) and a medium effect size in enamel (ES d*_Cohen_* = 0.77; Table 3, line 1). This result was mainly observed in the groups with the conventional nano-hybrid composite, as reflected by the statistically significant difference and large effect size (ES d*_Cohen_* = 2.27) between the NANO-METAL and NANO-TRANS groups (P_u_ = 0.0010 **) (Table 4 and Figure 5). However, the bulk-fill groups (BFC-METAL and BFC-TRANS) had no statistically significant difference between the two matrix types (P_u_ = 0.27).

Bulk-fill composite combined with the bulk-fill technique resulted in significantly more continuous margins within dentin (Table 3; P_u_ = 0.031 *, medium effect size d*_Cohen_* = 0.58). On the other hand, the quality of margins located within enamel did not differ significantly between the two composite materials or restorative techniques (P_u_ = 0.87) (Table 3).

## 4. Discussion

The aim of this study was to test the hypothesis that transparent matrices result in more continuous margins of bulk-fill composite restorations than metal matrices. The hypothesis was rejected, as no statistically significant difference in marginal quality between the two matrix systems could be detected. These findings are in agreement with those of other (laboratory and clinical) studies comparing transparent and metal matrices [27,34,41,42,43,44].

However, in the present study, the conventional nano-hybrid composite (Tetric EvoCeram) achieved significantly better marginal quality when applied using a metal matrix. This finding is in accordance with that of three older trials [26,30,35]. One explanation for this could be that the access cavity to the proximal box was smaller than the size of the light guide tip and thus blocked some of the polymerization light when the metal matrix was used [7,31]. This may have reduced the shrinkage stress of Tetric EvoCeram [45,46,47,48], resulting in fewer marginal gaps [35,49,50,51,52]. Whether this resulted in a lower depth of cure (DC) remains unclear as curing depth was not assessed in the present study. However, the three-sited light-curing technique was performed to achieve the best possible polymerization. Nevertheless, the data of Alshaafi et al. [7] and Price et al. [31] suggest that the depth of cure decreases if the tip of the light guide is partially covered, as might be the case when using a metal matrix. In the case of Tetric EvoCeram Bulk Fill, this effect might be less strong because its more efficient photo initiator makes polymerization of the material less susceptible to reduced radiant exposure while maintaining its physical properties and a sufficient depth of cure [11,15,53,54]. Therefore, we conclude that matrix type does not have such a strong influence on marginal gap formation with this bulk-fill composite.

Another explanation for the metal matrix resulting in higher proportions of perfect margins, especially with the conventional nano-hybrid composite (Tetric EvoCeram), might be that its reflective surface may have concentrated the polymerization light within the cavity, thus achieving a better depth of cure in deeper areas of the restoration [26]. With a transparent matrix, on the other hand, more light can exit the tooth and, therefore, less light reaches the deeper areas of the proximal boxes, resulting in poorer curing and poorer marginal quality. This assertion cannot be proven by measurements of the present study and may be subject to future studies. However, the findings by Kays et al. [26] suggest such an effect. Although we performed three-sited light curing after matrix removal to compensate for this, it must be assumed that the adjacent teeth of the artificial dental model and the hard tissue of the sample tooth itself attenuate light intensity when curing the buccal and lingual surfaces [35,55]. Conversely, the bulk-fill material could still be better polymerized than the conventional nano-hybrid composite due to its more efficient photo initiator. Nevertheless, there was a detectable, albeit not statistically significant tendency towards metal matrices resulting in better marginal quality in deeper areas of bulk-fill composite restorations (Figure 5).

Finally, this study is also subject to some methodological limitations, which must be discussed. First, artificial aging was achieved by performing 2500 cycles of thermocycling (5–55 °C), which is a rather short treatment period. Furthermore, the specimens were not loaded in a chewing simulator. However, a clear effect of the artificial aging protocol can be seen when looking at the proportions of continuous restoration margins and marginal gaps. This is supported by data from Frankenberger and Tay [56] and Peutzfeldt et al. [57], who observed marginal gap formation using either the same artificial aging protocol [56] or one with even fewer thermal cycles [57]. Nevertheless, it cannot be excluded that Tetric EvoCeram might have performed worse with the metal matrix due to a lower depth of cure, if more thermocycles or mechanical loading had been applied. However, in view of the large effect sizes (ES d*_Cohen_*) found in the present study, it is likely that a longer artificial aging period would have affected marginal gap formation in all other test groups as well, and that the relations between the test groups would have remained the same.

Another limitation of this study is that two materials from the same manufacturer were used. On the other hand, the two materials can be compared well with each other, as they are similar in terms of filler geometry and organic matrix. The results of the present study show that it might be worthwhile to conduct further studies on this research question with other materials.

Furthermore, flat matrix tapes were used in the present study because this was the easiest way to seal the cavity in this specific artificial dental model. Although these bands were used in other studies [22,23,24,25,58], there is consensus in literature that pre-contoured matrices (sectional or circumferential) are superior in clinical situations, especially for creating interproximal contacts and profiles [16,17,18,19,22,59]. In preliminary tests of various matrix systems (pre-contoured, sectional and circumferential), we ultimately selected the flat matrix bands as the preferred matrix system for reasons of practicality, i.e., because the focus of the present study was marginal gap formation rather than proximal contact tightness.

## 5. Conclusions

Taking into account the limitations of this study, it can be concluded that metal matrices have a positive influence on the marginal quality of deep class II composite restorations, and that this effect is more pronounced with conventional composite than with bulk-fill composite. Moreover, our findings indicate that bulk-fill composite achieves better marginal quality in deep cavity areas, and that its marginal quality is less sensitive to influence from factors such as light polymerization and the matrix system.

## Figures and Tables

**Table 1:** Material compositions and physical properties.

	Tetric EvoCeram ^a^		Tetric EvoCeram Bulk Fill ^b^	
Organical matrix [wt%]	Bis-GMA Bis-EMA UDMA	16.8	Bis-GMA Bis-EMA UDMA	19.7
Fillers [wt%]	Aluminoborosilicate glass, Ytterbiumtriflourid, Mixed oxides	48.5	Aluminoborosilicate glass, Ytterbiumtriflourid, Mixed oxides	62.5
	Prepolymers	34.0	Prepolymers	17.0
	Additives	<0.8	Additives	<1.0
Phototinitiators	Lucirin^®^-TPO Camphorquinone		Ivocerin^®^ Lucirin^®^-TPO Camphorquinone	
Flexural strength [MPa]	120		120	
Flexural modulus [MPa]	10,000		10,000	
Water absorption [μg/mm^3^], 7d	21.2		24.8	
Water solubility [μg/mm^3^], 7d	<1.0		<1.0	
Radio opacity [% Al]	400 (except for Bleach)		260	
	200 (Bleach I)			
	300 (Bleach L, M, XL)			
Depth of cure [mm]	>1.5		4	
Translucency [%]	6.5–20.0		14.0–16.0	
Vickers hardness HV 0.5/30 [MPa]	580		620	

Abbreviations: Bis-GMA, bisphenolglycidyl methacrylate; Bis-EMA, bisphenolglycidyl ethyl-methacrylate; UDMA, urethane dimethacrylate; TPO, Diphenyl (2,4,6-trimethylbenzoyl)-phosphine oxide. Materials compositition according to manufacturer’s scientific documentation from ^a^ February and ^b^ October 2011.

**Table 2:** Percentages of continuous margins in enamel and dentin (*n* = 40).

Continuous Margins [%]								
Margin Location	Mean	SD	Median	68%-CI		P_w_	d*_Cohen_*	ES s-m-l
				Lower	Upper			
				CI	CI			
Enamel	46.125	25.962	45.194	20.589	70.414	0.00005 ***	0.78	m
Dentin	22.577	24.349	15.674	0	42.839			
Total	36.642	20.412	33.366	20.685	58.413	-	-	-

P_w_ from Wilcoxon test, *** *p* < 0.001; ES, effect size d*_Cohen_*; s, small effect (d*_Cohen_* < 0.5); m, medium effect (d*_Cohen_* = 0.5–0.8); l, large effect (d*_Cohen_* > 0.8); CI = confidence interval; NANO = Tetric EvoCeram; BFC= Tetric EvoCeram Bulk Fill; METAL = metal matrix; TRANS = transparent matrix; CT = centripetal technique; BFT = bulk-fill technique.

**Table 3:** Pairwise comparisons of the four test groups (*n* = 20 each) according to the parameter matrix type and composite material (filling technique) in enamel and dentin; continuous margins [%] (*n* = 20 per group).

		**Continuous Margins [%]**							
**Margin** **Location**	**Matrix**	**Mean**	**SD**	**Median**	**68%-CI**		**P_u_**	**d*_Cohen_***	**ES** **s-m-l**
					**Lower**	**Upper**			
					**CI**	**CI**			
Enamel	METAL	55.491	22.477	54.067	27.141	75.203	0.013 *	0.77	m
	TRANS	36.759	26.337	37.836	12.359	50.353			
Dentin	METAL	32.349	25.155	25.902	8.135	50.120	0.0038 **	0.87	l
	TRANS	12.804	19.573	5.981	0.000	32.134			
Total (enamel + dentin)	METAL	46.211	14.912	47.799	33.028	63.291	0.0011 **	1.052	l
	TRANS	27.073	20.978	27.553	9.899	33.477			
**Margin** **Locatio**n	**Composite** **(Filling** **Technique)**	**Mean**	**SD**	**Median**	**68%-CI**		**P_u_**	**d*_Cohen_***	**ES** **s-m-l**
					**Lower**	**Upper**			
					**CI**	**CI**			
Enamel	NANO (CT)	46.231	29.005	47.021	16.359	71.126	0.87	-	-
	BFC (BFT)	46.019	23.286	43.859	26.790	66.368			
Dentin	NANO (CT)	15.716	20.832	6.636	0.000	32.192	0.031 *	0.58	m
	BFC (BFT)	29.437	26.151	23.865	7.198	44.914			
Total (enamel + dentin)	NANO (CT)	34.482	21.894	31.806	10.026	59.518	0.56	-	-
	BFC (BFT)	38.802	19.132	33.366	24.686	53.162			

P_u_ from Mann–Whitney U-test, * *p* < 0.05, ** *p* < 0.01; ES, effect size d*_Cohen_*; s, small effect (d*_Cohen_* < 0.5); m, medium effect (d*_Cohen_* = 0.5–0.8); l, large effect (d*_Cohen_* > 0.8); CI = confidence interval; NANO = nanohybrid composite (Tetric EvoCeram); BFC = bulk-fill composite (Tetric EvoCeram Bulk Fill); METAL = metal matrix; TRANS = transparent matrix; CT = centripetal technique; BFT = bulk-fill technique.

**Table 4:** Percentage of continuous margins [%] by margin location (enamel or dentin), composite material and matrix type (*n* = 10 per group).

	Groups	Continuous Margins [%]							
Margin Location	Composite–Matrix	Mean	SD	Median	68%-CI		P_u_	d*_Cohen_*	ES s-m-l
					Lower	Upper			
					CI	CI			
Enamel	NANO–METAL	65.935	22.724	66.863	48.644	86.094	0.0017 **	1.844	l
	NANO–TRANS	26.526	19.921	29.178	2.768	46.963			
Dentin	NANO–METAL	26.644	23.438	22.863	3.438	47.188	0.021 *	1.212	l
	NANO–TRANS	4.789	10.072	0.000	0.000	6.971			
Total (E + D)	NANO–METAL	50.857	15.947	51.389	36.907	66.868	0.0010 **	2.270	l
	NANO–TRANS	18.107	12.720	22.809	2.451	29.456			
Enamel	BFC–METAL	45.047	17.545	46.335	26.909	61.575	0.91	-	-
	BFC–TRANS	46.991	28.894	43.812	23.423	74.805			
Dentin	BFC–METAL	38.054	26.723	36.196	15.584	52.186	0.064	-	-
	BFC–TRANS	20.819	23.760	10.797	4.073	34.958			
Total (E + D)	BFC–METAL	41.564	12.929	41.039	29.364	51.575	0.27	-	-
	BFC–TRANS	36.040	24.261	28.686	16.419	56.898			

P_u_ from Mann–Whitney U-test, * *p* < 0.05, ** *p* < 0.01, ES, effect size d*_Cohen_*; s, small effect (d*_Cohen_* < 0.5); m, medium effect (d*_Cohen_* = 0.5–0.8); l, large effect (d*_Cohen_* > 0.8); CI = confidence interval; NANO = Tetric EvoCeram applied in centripetal technique; BFC = Tetric EvoCeram Bulk Fill applied in bulk-fill technique; METAL = metal matrix; TRANS = transparent matrix; E = enamel; D = dentin.

## Data Availability

Not applicable.
